# Herbal medicines adulteration with erectile dysfunction pharmaceuticals in sub-Saharan Africa: call to strengthen regulatory measures

**DOI:** 10.3389/fmed.2025.1694833

**Published:** 2025-11-05

**Authors:** Kampadilemba Ouoba, Daniel Dori, Adela Ashie, Issiaka Soulama, Rasmané Semdé

**Affiliations:** ^1^Laboratory of Drug Development, Joseph Ki-Zerbo University, Ouagadougou, Burkina Faso; ^2^National Pharmaceutical Regulatory Agency (ANRP), Ministry of Health, Ouagadougou, Burkina Faso; ^3^Food and Drugs Authority (FDA), Accra, Ghana; ^4^Institute for Research in Health Sciences (IRSS), Ouagadougou, Burkina Faso

**Keywords:** herbal medicines, adulteration, safety surveillance, regulation, sub-Saharan Africa

## Abstract

Sub-Saharan Africa is experiencing record levels of informal use of herbal medicines by the population for primary health care, with prevalence rates of up to 90% in some countries. This situation is linked to the high cost of pharmaceuticals and the popular perception that natural remedies are harmless. Furthermore, the proportion of men suffering from erectile dysfunction in sub-Saharan African countries remains high, varying between 25 and 70%. This dual challenge is at the root of the practice of adulterating herbal medicines with phosphodiesterase 5 inhibitors indicated for the treatment of erectile dysfunction, using abnormally high doses. This fraudulent practice poses a significant threat to consumer health, particularly for patients with comorbidities – potentially fatal erectile dysfunction, serious cardiac side effects, renal or hepatic failure, acute poisoning. This scourge is exacerbated by the weakness of regulatory systems in sub-Saharan African countries, especially their pharmacovigilance systems, which are still generally at low levels of maturity according to the WHO GBT. To combat this scourge, African countries must include the fight against pharmaceutical adulteration of herbal medicines in their policies and regulations on herbal medicines. Through their national regulatory authorities, they must develop and integrate the monitoring of herbal medicines safety into their national pharmacovigilance systems, formally involving herbalists, naturopaths and consumers. They must also develop and implement training programmes for herbalists and naturopaths in good phytotherapy practices, establish appropriate risk communication mechanisms, and ensure regular quality control of so-called natural health products sold on legal and illegal African markets, while tightening sanctions. In short, the fight against counterfeit herbal medicines must be fully integrated into the overall fight against counterfeit medicines in sub-Saharan Africa.

## 1 Introduction

There is generally high use of herbal medicines (HMs) in sub-Saharan Africa by the population for primary health care (1–3). The main contributing factors are the inadequacy of access to conventional healthcare due to the failure of healthcare systems in terms of infrastructure and human resources (3). There is also very limited financial and geographical accessibility to essential pharmaceutical products, leading to unprecedented inequalities in healthcare in this region (3, 4). Furthermore, the use of HMs, deeply rooted in culture, is accessible and affordable, and constitutes the main means of healthcare for a large part of the African population. It is firmly based on empirical herbal medicine and popular African beliefs that natural remedies are harmless to health, as nature is considered harmless (4, 5).

This context has led many African countries to develop national policies and regulatory frameworks for the promotion of traditional medicine (TM) in general and African herbal medicine in particular (5, 6). These policies and regulations aim to achieve the formal integration of traditional herbal medicine into African health systems, in order to contribute to the achievement of ‘health for all’, while protecting populations from related health risks (7). In many African countries, these initiatives have led to the creation of institutional frameworks for TM and the development of regulations governing the practice of TM, the opening up of traditional health establishments and procedures for registering HMs (8, 9). In the same vein, the WHO has published several guidelines for quality assurance of HMs in the African Region, including guidelines for HMs registration in the African Region, guidelines for HMs quality assessment and guidelines for HMs good manufacturing practice (10). Despite these efforts, the regulatory requirements governing TM in Africa are very rarely applied by traditional health practitioners (THPs), who remain the main players in TM (6, 11, 12). Furthermore, African countries do not have sustainable domestic funding for research into African herbal medicine, resulting in a serious lack of evidence on the efficacy and safety of HMs sold on African markets (13, 14).

These shortcomings, combined with the popular perception of the safety of natural plant-based remedies and the weakness of pharmacovigilance systems in sub-Saharan Africa, are conducive to the emergence of risks associated with African herbal medicine, especially in the precarious socio-economic conditions of the populations concerned (5, 12, 15). A number of studies have reported risks associated with the use of HMs, including adverse effects, toxicity, harmful pharmacokinetic or pharmacodynamic herb-drug interactions, microbial or heavy metal contamination and adulteration (16, 17). Adulteration in herbal medicine is a falsification practice that generally involves the intentional addition of conventional drugs or their active substances, or of one or more other plants, to herbal preparations, with the aim of fraudulently obtaining or increasing their therapeutic efficacy (10, 16). In sub-Saharan Africa, the adulteration of herbal products with conventional drugs, especially those indicated for erectile dysfunction (ED), that is phosphodiesterase-5 inhibitors, constitutes the most worrying risk group for public health. This pharmacological class is particularly affected by the falsification of HMs due to the important prevalence of ED in African countries. Surveys of men between 34 and 70 y of age seeking primary medical care indicated that the age-adjusted prevalences of ED were 25.4% in Ethiopia, 57.4% in Nigeria and 63.6% in Egypt (18–20). This prevalence exceeds 70% among African diabetic patients (21). This generates a significant market for ED drugs; for example the Nigeria ED drugs market generated a revenue of USD 4.2 million in 2024 and is expected to reach USD 7.4 million by 2030 (22).

In addition to its epidemiology in Africa, ED is subject to social stigma, thus creating a psychological burden on those affected (20). These individuals generally resort to natural products with aphrodisiac properties because they are more affordable and accessible than synthetic aphrodisiac drugs and because they are perceived by the population as being safer for health than allopathic medicines (3). Although there are scientifically validated African aphrodisiac plants for treating ED (23), this situation encourages herbalists and naturopaths to adulterate their products by adding synthetic aphrodisiacs based on sildenafil or its analogues for economic reasons and due to the lack of systematic regulatory quality control (24). These adulterated products are then labelled ‘100% natural’ with disregard for consumer health.

The aim of this article was to highlight the public health threat posed by HMs adulteration with ED pharmaceuticals in sub-Saharan Africa.

## 2 Status of informal use of herbal medicines in sub-Saharan Africa

In countries with limited resources, the WHO estimates that more than 80% of the population relies on traditional and complementary medicine, particularly herbal medicine, for primary healthcare (9). In sub-Saharan Africa, the prevalence of people using HMs varies from 85% in Burkina Faso and Nigeria, 86% in Ghana, to 90% in Burundi, Ethiopia, and Sudan (3, 25–27). Annually, the trade of medicinal plants is estimated in US dollars at 117,612 in Mali, 2.2 million in South Africa and 5.37 million in Burkina Faso (9). In recent years, recourse to this treatment has increased in the context of epidemics or pandemics such as those involving COVID-19 and dengue fever. This is due to the lack of curative treatments for these re-emerging infections (15).

The medical conditions for which HMs are used mainly concern priority diseases in Africa, such as malaria, tuberculosis, HIV/AIDS, viral hepatitis, infectious childhood diseases and chronic non-communicable diseases such as diabetes, high blood pressure, cancer, kidney disease, etc. (3, 25). Patients suffering from these chronic diseases use phytotherapy in combination with conventional medicines, often without informing their doctors, which could cancel out or reduce the efficacy of the conventional treatment or potentiate one of its undesirable effects (3).

In addition, the instructions for use of HMs recommended by traditional healers for the use of their products (dosage, dosage materials, duration of treatment, precautions and contraindications) are not standardised and are based exclusively on the empirical knowledge and practices of African traditional medicine (11). A recent study showed that these practices largely failed to meet the regulatory requirements and good practices applicable to HMs, mainly due to a lack of training programmes for THPs, particularly herbalists and naturopaths (11).

Despite the perceived safety of traditional phytotherapy, high frequencies of adverse events due to HMs have been reported in African countries—10% among health professionals and 15% among the general population in Burkina Faso and 16% in Morocco (3). In Nigeria, Awodele et al. observed that traditional therapists are sometimes informed by their patients of the occurrence of adverse effects due to the herbal and traditional medicines they market, but that very few of them document them (28). The lack of reporting of adverse reactions or quality problems associated with HMs in African countries is a real problem. A recent assessment of pharmacovigilance systems in eight French-speaking West African countries revealed that HMs are not formally integrated into national health product surveillance systems and that there are no Community provisions for the regulatory surveillance of HMs. This is despite the public health threat posed by the unregulated use of HMs, especially as regards their adulteration in this region (5).

## 3 Critical condition of herbal medicines adulteration with erectile dysfunction pharmaceuticals in sub-Saharan African countries

It is generally estimated that more than 500,000 people die each year in sub-Saharan Africa as a result of consuming dangerous and unregulated medicines, including products intended to stimulate male libido (29). The most recent case concerns the HMs adulteration and other “natural products” reputed to be aphrodisiacs, identified in Côte d’Ivoire, West Africa (30). On September 2, 2024, the Ivorian Pharmaceutical Regulatory Authority (AIRP) issued an information note concerning the suspension of the import and marketing of a list of eight so-called 100% natural aphrodisiac products. These products claimed to be organic or natural include ‘*Vitamax doubleshot Energy coffee box of 10 sachets’*; ‘*Vitaplus Cacao power box of 12 sachets’*; ‘*Vitamax kingsman energy candy box of 12 pastilles’*; ‘*Bio herbs coffee powder box of 8 sachets’*; ‘*Bio herbs royal king honey box of 10 sachets’*; ‘*Maximen Bio Honey oral solution box of 10 sachets’*; ‘*Cappuccino Maximen coffee box of 10 sachets’* and ‘*Kopivitamine box of 8 sachets’*. This regulatory decision follows quality control tests carried out by Côte d’Ivoire’s National Public Health Laboratory (LNSP), which revealed the presence of Tadalafil. Tadalafil is a synthetic drug indicated for the treatment of ED in adult men, which requires a doctor’s prescription, especially for patients with cardiovascular risk factors. This decision to ban the marketing of these fake natural products has also been taken by the National Drug Regulatory Authorities (NRAs) of Bénin and Burkina Faso, where these products are widely marketed and consumed by the general population.

As early as April 2024, the AIRP carried out a quality control on so-called natural products reputed to be aphrodisiacs called ‘*Attote original 100% naturel*’ and ‘*La paix cognons-mousso-yako*’. These products do not have marketing authorisations but are widely sold on the informal market in Côte d’Ivoire and other West African countries (30). Quality control tests carried out by Côte d’Ivoire’s LNSP on samples of these products revealed the presence of large quantities of sildenafil, a drug indicated for the treatment of ED. Consumption of these products adulterated with sildenafil can lead to headaches and dizziness in patients with hypertension or cardiovascular risks, strokes, heart attacks and even sudden death. In view of these public health risks, the AIRP has issued an information note urging people to refrain from consuming the incriminated products and their derivatives, and has decided to suspend their manufacture and marketing in Côte d’Ivoire.

In other West African countries, such as Burkina Faso, the consumption of natural plant-based products designed to improve sexual performance is on the increase, while at the same time encouraging the practice of adulteration for purely economic reasons. It was against this backdrop that, in 2020, Burkina Faso’s LNSP undertook quality control of herbal preparations for ED sold in the capital, Ouagadougou (31). A total of 44 samples were collected and the analytical results revealed that 10 samples from the informal market (22.7%) were adulterated with sildenafil (80%) and tadalafil (20%). The quantities of sildenafil detected ranged from 2.6 mg to 108.2 mg, which is up to more than five times the usual recommended dose of 20 mg. For tadalafil, the quantity measured per daily sample dose was 37.6 mg, nearly four times the typical recommended dose of 10 mg (31). These results show that the HMs and other natural health products sold on African informal markets are still not as natural as their operators claim, and that regulatory initiatives need to be taken to protect public health from the risks associated with this adulteration and concealment.

In Bénin, a study to identify falsifications of ED pharmaceuticals in plant-based products reputed to be aphrodisiacs was carried out in the communes of Cotonou and Abomey-Calavi, in order to estimate the scale of the problem (32). To this end, 77 herbal aphrodisiacs were identified and analysed. Regulatory analysis revealed that the majority of these products (87%) did not comply with labelling requirements. Analytical tests showed that 33% were adulterated with tadalafil, with concentrations ranging from 1.7 to 4.6 times higher than the normal recommended dose (32).

In Mali in 2024, a study screening for phosphodiesterase 5 inhibitors in natural aphrodisiacs marketed in the Bamako district was conducted by the National Health Laboratory. Twenty-two samples underwent identification tests using thin-layer chromatography (TLC) and Fourier transform infrared spectroscopy (FTIR). The majority of the samples tested were adulterated with phosphodiesterase 5 inhibitors. Of the 22 samples, two tested positive for sildenafil, two for tadalafil, and 14 for both by FTIR. However, five tested positive for sildenafil, nine for tadalafil, and four for both by TLC; representing 82% (18/22) of adulterated samples for each test (33).

In Kampala, Uganda (East Africa), a survey of HMs sold in street markets and pharmacies showed that more than half (54%) of the products analysed were adulterated with ED pharmaceuticals, including sildenafil, tadalafil and vardenafil (24). The adulteration of HMs with phosphodiesterase-5 inhibitors to improve sexual performance is a serious public health threat in several African countries (34). Just like counterfeit medicines, the use of HMs adulterated with synthetic aphrodisiacs can negatively affect all aspects of users’ lives—death or other serious adverse effects, chronic diseases such as chronic renal, hepatic or cardiac failure, treatment failures, loss of confidence in licensed herbalists practising their profession in accordance with regulations, loss of revenue for legal manufacturers, and finally, corruption of the medicinal plant supply chain (35).

The addition of active pharmaceutical ingredients for ED to so-called ‘natural’ medicinal products is a serious public health problem that has worsened in recent years in sub-Saharan Africa. The cases described above may only be the tip of the iceberg. It is therefore imperative to strengthen regulation, surveillance and quality control by NRAs in sub-Saharan African countries to stem this growing threat.

## 4 Future direction for countering the threat

Continental organizations such as the African Medicines Agency (AMA), Africa CDC and the WHO/African Region and regional economic communities must consider HMs as public health priority, in view of their popular use by the population. They should develop continental guidelines for the regulation and safety monitoring of HMs and encourage Member States to formally integrate them into their pharmacovigilance systems.

The AMA, through the AUDA-NEPAD Vigilance Technical Committee (VTC), should develop continental guidelines for safety monitoring of HMs, taking into account risk communication and quality control. These guidelines should be integrated into those for the monitoring of medical products in general, so as not to duplicate efforts. Given that VTC members are representatives of the African Union’s Regional Economic Communities (RECs), these guidelines could be easily implemented by these communities.

Africa CDC, through its Science and Innovation Directorate, should implement applied research to help ensure the safety of herbal medicinal products on the continent.

These continental organisations should then provide technical assistance to RECs to drive the implementation of continental guidelines and promote applied research on medicinal plants and HMs at the regional level. REC should in turn provide technical assistance to their member countries to help them adopt the continental guidelines. At the national level, in addition to the application of continental guidelines, the main recommendation for combating the HMs adulteration with pharmaceuticals generally in sub-Saharan Africa must be the formal integration of this scourge into HMs policies and regulations. At the political level, African states must define national strategies to combat the falsification of HMs, incorporating them into existing TM policies. These strategies must involve all the stakeholders, i.e., the ministry in charge of health through its TM directorate or programme, the NRAs, the decentralized structures of the health system, the federations and associations of THPs and local authorities (12). They must also determine a sustainable method of internal funding for research and development of medicines derived from traditional pharmacopoeia. This should take into account funding for quality control of HMs available on the legal and illicit African markets (5).

On the subject of regulation, countries must adapt the regulatory frameworks governing the practice of TM and HMs to their socio-cultural contexts. Studies have shown that the current regulations on African TM are very orthodox and poorly adapted to the socio-cultural contexts of African countries, hampering their application by TM players (6, 8). Contextualized regulatory frameworks could therefore encourage collaboration between traditional and conventional medicines, promote the registration of HMs and reduce falsification practices in herbal medicine (6).

The other key prospect is the development of phytovigilance, the aim of which is to monitor the safety and risks associated with plant-based medicinal products (36). The NRAs of sub-Saharan African countries must consider phytovigilance as a public health priority (37). In view of the limited resources available in these countries, phytovigilance must be integrated into existing national pharmacovigilance systems, so as not to duplicate efforts (5). Also, given the high level of informal use of HMs in these countries, it is essential that THPs and consumers are involved in the reporting systems for HMs adverse effects, with reporting materials designed in the main local languages of the countries concerned (5, 15). Sustainable initiatives must also be taken in terms of communication, awareness-raising, training and education of THPs and the general public about the risks associated with the informal use of HMs. National training programmes on WHO good practice in HMs should be designed and implemented specifically for THPs.

Finally, in the wake of the creation of NRAs in sub-Saharan Africa, countries must create or strengthen their drug quality control laboratories (37). Special attention should be paid to regular quality control of so-called natural products for ED treatment sold on informal markets in Africa. This will enable the detection of any possible adulteration with pharmaceutical drugs and the application of dissuasive sanctions. In short, to be more effective, the fight against the falsification of HMs must be integrated into the overall fight against falsified medicines in sub-Saharan Africa.

Other entities such as universities should also contribute to the fight against pharmaceutical adulteration of HMs, in particular by conducting research to develop less costly methods for detecting adulterants and contaminants in herbal medicinal preparations.

Taking the example of Burkina Faso, a French-speaking country in West Africa, the table above describes the current regulatory framework and the actions that should be strengthened.

**Table 1:** 

Field	Reference and provisions	Actions to strengthen
Policy	National Strategy Framework for Traditional Medicine and Pharmacopoeia	Include the monitoring of herbal medicines safety in the strategy
Legislation	Act No. 23/94/ADP of 19 May 1994 establishing the Public Health Code official recognition of traditional medicine official recognition of the profession of traditional health practitioner consideration of medicinal plants promotion of research into traditional pharmacopoeia	Taking herbal medicines into account in health vigilance
Regulatory framework	Decree No. 2004-569/PRES/PM/MS/MCPEA/MECV/MESSRS authorising the marketing of medicines derived from traditional pharmacopoeia Order No. 2013-552/MS/CAB laying down the terms and conditions for the practice of traditional medicine in Burkina Faso Order No. 2013-550/MS/CAB on the terms and conditions for opening and operating a traditional medicine and pharmacopoeia establishment in Burkina Faso Order No. 2013-551/MS/CAB on the establishment, organisation and operation of a referral and appeal system between traditional medicine and conventional medicine in Burkina Faso	Strengthen regulatory inspections
Vigilance system	Decree No. 2018-0911/PRES/PM/MINEFID/MS approving the specific statutes of the National Pharmaceutical Regulatory Agency Decree No. 2012-1033/PRES/PM/MS establishing the creation, powers and organisation of a national vigilance system for health products for human use Order No. 2013-542/MS/CAB establishing the reporting procedure for adverse effects of health products	Formally include traditional medicines and herbal medicines in the reporting system Formally involve traditional health practitioners and consumers in the reporting system Conduct surveys on the quality of herbal medicines on the market Conduct quality control on herbal medicines reputed to be natural Raise awareness among the general public Promote reporting by healthcare professionals and consumers

## 5 Discussion

The populations of sub-Saharan Africa make extensive informal use of HMs, due to the inability of conventional healthcare systems to meet their healthcare needs and to their therapeutic traditions, which are deeply rooted in African herbal medicine. This unregulated use encourages health risks and the emergence of dangerous practices such as the adulteration of herbal medicinal products with pharmaceuticals. This represents a serious threat to public health and hampers the integration of traditional phytotherapy into modern health systems in Africa.

For African countries, this scourge must be a public health priority to be taken into account in national TM policies and regulations. NRAs must work to put in place regulatory frameworks that are adapted to the cultural contexts of countries and that promote open collaboration between traditional and conventional medicines. They must also develop and integrate phytovigilance into national pharmacovigilance systems, with the legal involvement of herbalists, naturopaths and consumers.

The design and implementation of national programmes to train herbalists and naturopaths in good practice applicable to HMs, appropriate communication mechanisms aimed at the general public on the risks associated with the informal use of HMs and regular quality control of so-called natural products, based on dissuasive measures, are the main levers in the fight against the adulteration of HMs in sub-Saharan Africa. These measures to combat the falsification of HMs must be integrated into the overall fight against falsified medicines in sub-Saharan Africa.

## Data Availability

The original contributions presented in the study are included in the article/supplementary material, further inquiries can be directed to the corresponding author.
